# Choosing and switching biologics for patients with severe asthma: Real-world data from the German Asthma Net (GAN)

**DOI:** 10.5414/ALX02636E

**Published:** 2026-07-20

**Authors:** Alexandra Lenoir, Roland Buhl, Carlo Muemmler, Jürgen Behr, Rainer Ehmann, Eckard Hamelmann, Anette Holtdirk, Marco Idzko, Margret Jandl, Frank Kaessner, Olaf Schmidt, Christian Schulz, Dirk Skowasch, Hendrik Suhling, Christian Taube, Stephanie Korn, Katrin Milger, GAN investigators

**Affiliations:** 1 Department of Medicine V, LMU University Hospital, Comprehensive Pneumology Center (CPC), Member of the German Center of Lung Research (DZL), LMU Munich, Munich,; 2 Mainz University Hospital, Pulmonary Department, Mainz,; 3 Ambulante Pneumologie mit Allergiezentrum, Stuttgart,; 4 Kinderzentrum Bethel, Evangelisches Klinikum Bethel, University Bielefeld, Bielefeld,; 5 RQMplus Germany, Ahlen, Germany,; 6 Department of Pneumology, University Hospital Vienna AKH, Medical University of Vienna, Vienna, Austria,; 7 Hamburger Institut für Therapieforschung GmbH, Hamburg,; 8 Ambulantes Zentrum für Lungenkrankheiten und Schlafmedizin, Cottbus,; 9 Pneumologie Mittelrhein und Studienzentrum KPPK, Bendorf am Rhein,; 10 Clinic and Polyclinic of Internal Medicine, Department of Pneumology, University Hospital Regensburg, University of Regensburg, Regensburg,; 11 Department of Internal Medicine II - Pneumology, University Hospital Bonn, Bonn,; 12 Pneumologicum Hannover, Hanover,; 13 Department of Pulmonary Medicine, University Hospital Essen-Ruhrlandklinik, Essen,; 14 IKF Pneumologie Mainz and Thoraxklinik Heidelberg, Mainz and Heidelberg, Germany,; 15 Division of Respiratory Medicine, Department of Internal Medicine, Lung Research Cluster, Medical University of Graz, Graz, Austria, and; 16 Institute of Lung Health and Immunity (LHI), Comprehensive Pneumology Center (CPC), Helmholtz Munich, Member of the German Center of Lung Research (DZL), Munich, Germany

**Keywords:** asthma, biologics, asthma treatment, biomarkers, eosinophils

## Abstract

With six biologics available to treat severe asthma in 2025, choosing the optimal initial therapy and deciding to which other biologic to switch in case of insufficient benefit is challenging. To better understand patients’ characteristics depending on the chosen biologic and patterns of switch, we evaluated adult patients with severe asthma from the GAN registry who were biologic-naïve at baseline. Between 2011 and 2024, 2,649 patients with severe asthma newly received a biologic, 26% anti-interleukin-5 receptor (IL5R), 25% anti-IL5, 16% anti-IL4R, 24% anti-immunoglobulin E (IgE), and 9% anti-thymic stromal lymphopoietin (TSLP). Distribution of biologics varied between time periods, reflecting their availability over time. Patients treated with anti-IgE were youngest at asthma diagnosis (mean 26 years) and had the highest rates of allergic comorbidities (80%). Blood eosinophils were highest in patients receiving anti-IL5R (median 433/µL) and lowest in those receiving anti-TSLP (median 159/µL), while FeNO was highest in patients treated with anti-IL5, anti-IL5R, or anti-IL4R (median 39, 38, and 35 ppb, respectively). 14.3% (n = 378) of patients were switched from the first biologic to a different one. The most frequent switch constellations were anti-IL5 to anti-IL5R, anti-IL5R to anti-IL4R, and anti-IL5 to anti-IL4R. After a switch, depending on the constellation up to 46% of patients reached remission. Phenotyping patients with severe asthma determines the choice of biologic therapy in real-world practice. Choice and switch of biologic have evolved over time influenced by availability of different drugs. Our analysis supports the practice of switching to a different biologic in case of insufficient initial response.

## Introduction 

Severe asthma is defined as asthma that requires high-dose inhaled corticosteroids and an additional controller, usually a long-acting β_2_-agonist (LABA), and/or systemic corticosteroids to remain controlled or remains uncontrolled despite this treatment [1, 2]. Its prevalence varies, depending on the studied population, from between 3% and 10% of all asthma patients [3, 4, 5]. Uncontrolled severe asthma comes with a high economic, health care, and quality of life burden [6, 7]. Over the last two decades up to 2025, six monoclonal antibodies have become available to treat severe asthma: the anti-immunoglobulin E (IgE) antibody omalizumab, the anti-interleukin 5 (IL5) antibodies mepolizumab and reslizumab, and the anti-interleukin-5 receptor (IL5R) antibody benralizumab, the anti-interleukin-4 alpha receptor (IL4R) antibody dupilumab, and the anti-thymic stromal lymphopoietin (TSLP) antibody tezepelumab, available in Germany since 2022. They target different parts of the type-2 inflammation pathways driving asthma [8] and have proved to achieve clinical response, i.e., reduction of exacerbations and/or of the dose of oral corticosteroids, and even remission in a relevant proportion of patients with previously uncontrolled severe asthma not only in controlled clinical trials but also in real-world settings [4, 9, 10]. However, as more biological therapies have become available for severe asthma, scientific data and clinical practice have shown that phenotyping and careful patient selection before starting a biologic is key to achieving the best possible result in asthma control [11, 12]. 

As of yet, there are no head-to-head comparisons in severe asthma beyond expert opinion to support respiratory physicians in the choice of biologic therapy [13, 14], making not only the choice of the first biologic a complex decision but also the switch from one biologic to another if no satisfactory asthma control could be achieved with the first. 

Therefore, our aim was to describe patient characteristics and their differences depending on the initially chosen biologic in real-world practice in Germany, a country where all six available biologics are covered by the statuary health insurance if EMA licensing criteria are fulfilled. Furthermore, we aimed to analyze prescription practice of asthma biologics over time, taking into account their availability, and to report frequency, timing and type of switch to a different biologic whenever occurring. Finally, we describe the change in asthma control and type 2-inflammation biomarkers depending on the respective switch constellations. 

## Materials and methods 

### Study population 

The German Asthma Net (GAN) is a registry collecting data from severe asthma centers in Germany and Austria since 2011, including secondary and tertiary hospital centers as well as specialized respiratory practices (germanasthmanet.de) [15, 16]. It includes structured information on diagnosis, treatment, comorbidities, and biomarkers of adult and pediatric patients with severe asthma. The participants’ diagnosis of severe asthma as by ERS/ATS criteria [2] is made by specialized respiratory physicians. Data is collected prospectively at baseline and through annual follow-ups. The GAN registry has been approved by the Ethics Committee of the University of Mainz and the local Institutional Review Boards and in accordance with the principles of the Declaration of Helsinki. All participating patients provided their informed consent prior to inclusion. 

### Inclusion criteria and definitions 

For the current study, we used data from patients aged 18 years and above at the baseline visit (V0), included until December 31, 2024 (date of data extraction) in the German registry sites. We included individuals who did not use biological asthma therapy at inclusion and were started on a biologic thereafter. The initial asthma biologic was defined as the first documented biologic in the registry with no evidence of prior biologic therapy. Asthma therapy including biologics was prescribed by the treating respiratory physician according to national and international guidelines [13, 17]. We included patients with available data on asthma control and lung function at V0 and follow-up, i.e., when started on the first biologic treatment. Due to the availability of different biologics, time periods for the initial biologic therapy were defined as follows: before 2015 (only omalizumab available in Germany), 2015 to 2018 (mepolizumab, benralizumab, and reslizumab became available), 2019 to 2022 (dupilumab became available), and after 2022 (tezepelumab became available). 

In a second step, we analyzed patients who had had a switch of biologic 1 year after that switch. If there was no follow-up data 1 year after a switch was available, data from 2 years after the switch was used. When follow-up data were available for neither 1 nor 2 years after a switch, data from the 4-months follow-up after the switch was used for analysis. We analyzed the change in exacerbation frequency, asthma control test (ACT) scores, use of oral corticosteroids (OCS), FEV_1_, response as by Biologic Asthma Response Score (BARS) including these four previous items [10], levels of fractional exhaled nitric oxide (FeNO) in parts per billion (ppb), absolute blood eosinophil count (/µL), and remission after change to a new biologic therapy. Remission I was defined as no exacerbations in the preceding 12 months, no OCS use, and ACT ≥ 20 points, remission II as all of the above as well as an FEV_1_ improvement of ≥ 100 mL. 

### Statistical analysis 

For the statistical analysis, the anti-IL5 antibodies mepolizumab and reslizumab were grouped into one category. Normal distribution was verified through Kolmogorov-Smirnov test. Categorical variables were compared using χ^2^, continuous variables did mostly not follow a normal distribution, and their medians were therefore compared using the Mann-Whitney U test or, if more than two groups were compared, the Kruskal-Wallis test. Changes after switching from the first to a new biologic therapy were analyzed using signed rank test for paired data. All statistical analyses were conducted using the statistical software SAS 9.4 for Microsoft Windows and applying a significance level (alpha) of 0.05. 

## Results 

**Figure 1.**
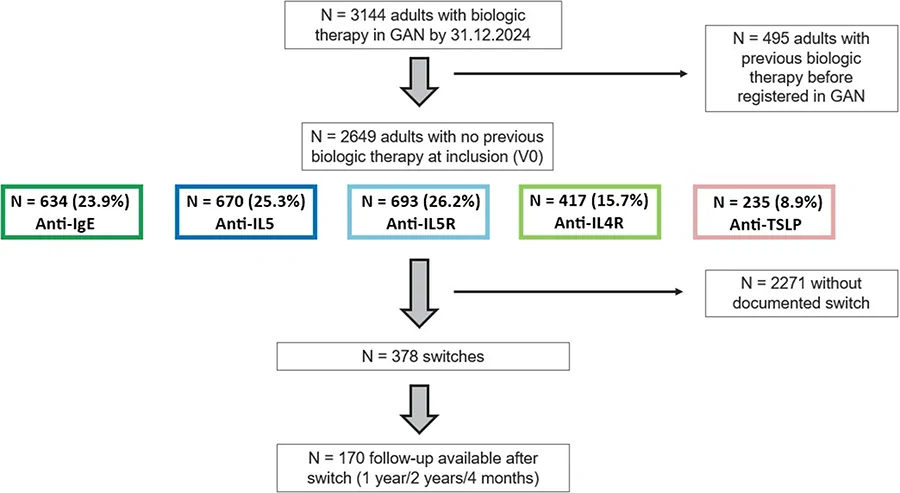
Inclusion flow chart for biologic therapies in severe asthma in the GAN registry 2011 – 2024. GAN = German Asthma Net; V0 = visit at inclusion; anti-IgE = anti-immunoglobulin E; anti-IL5 = anti-interleukin-5; anti-IL5R = anti-interleukin-5 receptor, Anti-IL4R = anti-interleukin-4 alpha receptor; anti-TSLP = anti-thymic stromal lymphopoietin.

In total, data from 3,144 adult patients with severe asthma treated with a biologic were available in the GAN registry on December 31, 2024, of whom 495 (15.7%) had already received a biologic therapy for asthma before inclusion in the registry and were therefore excluded from this study (Figure 1). Among the 2,649 patients who were initially biologic naïve, 378 (14.3%) were switched from the first biologic therapy to a different one. 

**Figure 2.**
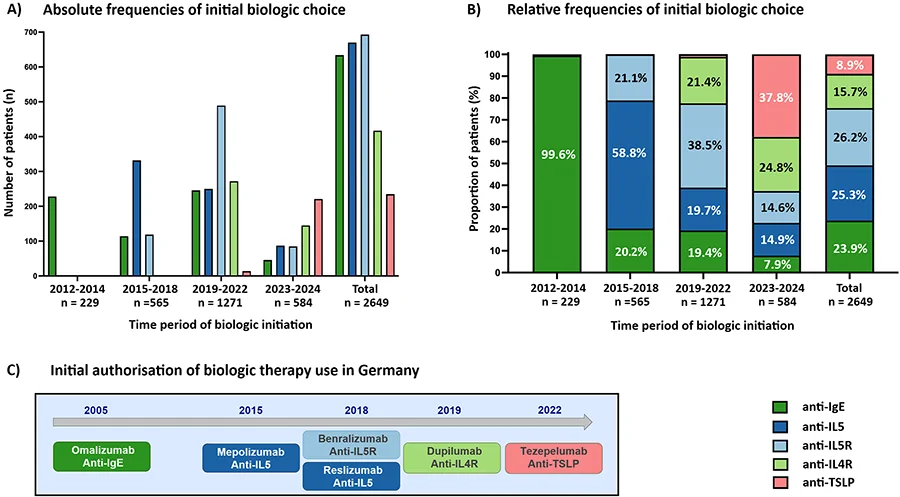
Initial biologic choice over time for severe asthma over time in the GAN registry 2011 – 2024. A: Absolute frequencies of initial biologic choice. B: Relative frequencies of initial biologic choice. C: Initial authorization of biologic therapy use in Germany. GAN = German Asthma Net; anti-IgE = anti-immunoglobulin E; anti-IL5 = anti-interleukin-5; anti-IL5R = anti-interleukin-5 receptor; anti-IL4R = anti-interleukin-4 alpha receptor; anti-TSLP = anti-thymic stromal lymphopoietin.

Figure 2 shows the frequency of new asthma biologic initiations over time. Whilst in the years 2012 – 2014 nearly exclusively anti-IgE (omalizumab) was started, this being the first available antibody for severe asthma, the picture became more balanced over the following years with subsequent approval of other antibody types. In the period between 2022 – 2024, the anti-TSLP tezepelumab represented the most frequently initiated asthma biologic. Of note, the absolute numbers of patients starting an antibody therapy increased over time, so that even though, e.g., the relative proportion of patients starting omalizumab decreased, absolute numbers remained stable until 2022 (Figure 2). 

Patients’ baseline characteristics depending on the first initiated asthma biologic are shown in Table 1. Patients who received anti-IgE were on average younger (mean 49.8 years) than patients who received other biologics. Patients treated with anti-IgE being the youngest at asthma diagnosis (mean 26.5 years), while anti-IL5 and anti-IL5R treated patients were the oldest (mean 41.2 and 41.0 years, respectively). Patients treated with anti-IgE, and to a lesser extent those treated with anti-IL4R, had higher rates of allergic comorbidities than the other groups (79.6% and 61.1%, respectively). Comorbid chronic rhinosinusitis and nasal polyps were more frequent in patients treated with anti-IL5, anti-IL5R, and anti-IL4R. 

Patients treated with anti-IgE had the lowest exacerbation rates in the preceding year (7.4% with ≥ 1 exacerbations/month), whilst patients started on anti-TSLP had the lowest proportion with no exacerbations in the preceding year (16.2%). This is also reflected in the GINA control status where patients started on anti-IgE had the highest proportion with controlled or partially controlled asthma, whilst patients started on anti-TSLP, anti-IL5R, and anti-IL4R had the highest proportion of uncontrolled GINA status and on average the lowest ACT score (median 14 points). Patients treated with anti-IgE had the highest FEV_1_ (mean 69.83% predicted). Patients treated with anti-IL5, anti-IL5R, and anti-IL4R had higher values for fractional exhaled nitric oxide (FeNO) than those treated with anti-IgE and anti-TSLP. Median blood eosinophils were highest in patients subsequently receiving anti-IL5R (433/µL) and lowest in patients receiving anti-TSLP (159/µL) at baseline. Total IgE was higher in patients who were subsequently treated with omalizumab than in the other groups (p < 0.001) (Table 1). The highest proportion of patients with systemic steroid treatment was found among those initiated on anti-IL5 and anti-IL5R (35.4% and 33.8%, respectively). 

**Figure 3.**
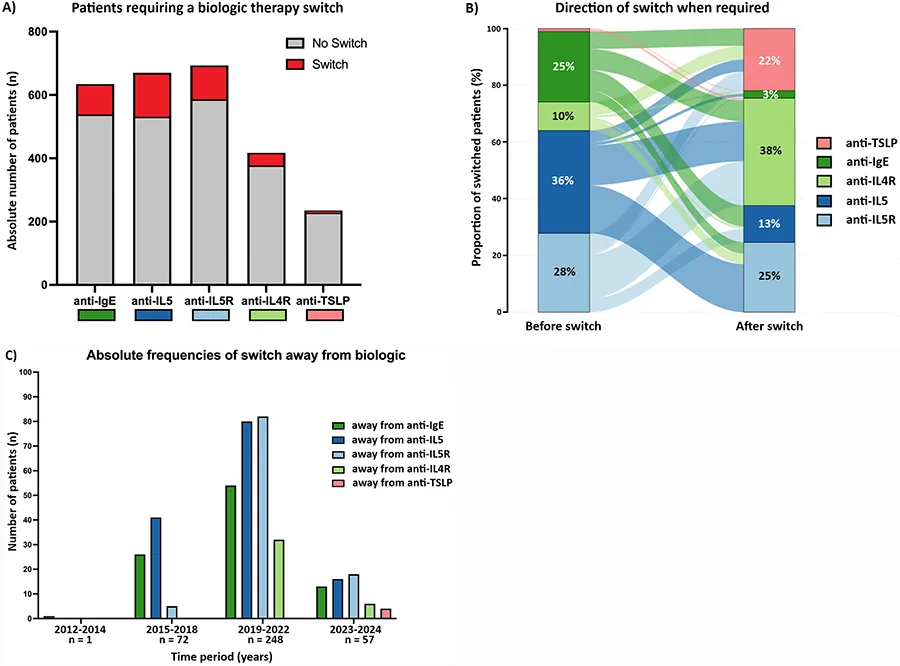
Number and direction of biologic therapy switch for severe asthma over time in the GAN registry 2011 – 2024. A: Patients requiring a biologic therapy switch. B: Direction of switch when required. C: Patients switching away from biologic according to time period. Total number of switching patients n=374, percentages are relative to this number. GAN = German Asthma Net; anti-IgE = anti-immunoglobulin E; anti-IL5 = anti-interleukin-5; anti-IL5R = anti-interleukin-5 receptor; anti-IL4R = anti-interleukin-4 alpha receptor; anti-TSLP: = anti-thymic stromal lymphopoietin.

Table 2 shows the exact switch constellations among the 378 patients whose treatment was changed from the first to a different biologic, and Figure 3 summarizes them graphically. Duration between start of the first biologic therapy and switch to a different one varied between the 5 antibody groups with earlier switch away from anti-TSLP (median after 10.9 months) and anti-IL4R (median after 14.4 months), and later switch away from anti-IgE (median after 19.9 months), anti-IL5R, and anti-IL5 the latest (median after 22.4 and after 24.2 months) (Table 2) (p = 0.029). Also, the numbers of patients who switched varied between different time periods, with the highest number of switches occurring in the period between 2019 – 2022 (n = 248) (Figure 3C). After the switch of biologic therapy, 170 patients had follow-up data available for analysis (Supplemental Table 1). Of note, not all switch constellations had available follow-up, for example, there was no follow-up information available for patients who switched from an older biologic to the most recently available anti-TSLP. 

Numbers of exacerbations in the last 12 months before and after antibody switch are shown in Supplemental Table 2. After switch from anti-IL5 to anti-IL5R therapy, patients had on average 0.5 ± 1.2 less exacerbations than in the preceding 12 months (p = 0.01), and after switching from anti-IL5R to anti-IL4R therapy, patients had 1.3 ± 2.9 less exacerbations than in the preceding 12 months (p = 0.006). Similarly, improvements in GINA control status and ACT were observed after several switch constellations (Supplemental Tables 3 and 4). There was no significant difference in FEV_1_ (liters) after any antibody switch constellation, however, a slight improvement in FEV_1_ (%predicted) after switch from anti-IL5 to anti-IL4R (mean +5.9% ± 13.5, p = 0.025) (Supplemental Tables 5 and 6). FeNO levels showed a median decrease of 20 ppb after switching from anti-IL5 to anti-IL4R (p = 0.002) and 16 ppb after switching from anti-IL5R to anti IL4R (p = 0.008) (Supplemental Table 7). Absolute blood eosinophil count increased with a median of 232 /µL after switching from anti-IL5R to anti-IL4R (p = 0.04) and tended to decrease with a median of 15/µL after a switch from anti-IL5 to anti-IL5R (p = 0.06) (Supplemental Table 8). There was no change in the proportion of patients receiving systemic steroids (p = 0.21) and no change in daily prednisolone equivalent dose (data not shown). 

After a switch, and depending on the exact switch constellation, between 79% and 90% of patients had an intermediate or good response to the new biologic therapy according to the BARS I score, and between 64% and 80% according to the BARS II score, the latter also accounting for lung function (Table 3). Similarly, after a switch, depending on the constellation, between 25% and 46% of patients fulfilled criteria for remission I (no exacerbations in the previous 12 months, no OCS use, and ACT ≥ 20 points), and between 6% and 23% fulfilled criteria for remission II (no exacerbations in the previous 12 months, no OCS use, ACT ≥ 20 points, and FEV1 improvement of ≥ 100 mL) (Table 4). 

## Discussion 

In this real-world, multicentric observational study, we show that the initial choice of biologic therapy in patients with severe asthma has changed over time in Germany between 2011 and 2024 as new antibodies have become available, with anti-IgE being exclusively initiated before 2015, then progressively joined by anti-IL5, anti-IL5R, anti-IL4R, and lastly anti-TSLP, which has become the most frequently chosen first biologic in the last 2 years. Alongside availability, our data also show that the choice of biologic corresponds to specific phenotypes, for example anti-IgE being usually initiated in patients with early-onset asthma and allergic comorbidities, whilst patients started on anti-IL5, IL5R, and anti-IL4R usually suffered from late-onset asthma, frequent chronic rhinosinusitis and nasal polyps, and had high FeNO levels. The recommendations to base the choice of antibody in severe asthma on a phenotype composed by age of onset, comorbidities, and biomarkers [13, 14] are therefore confirmed in our real-world data and seems to correspond to routine clinical practice. Interestingly, anti-TSLP was initiated in patients who had at baseline the lowest blood eosinophil counts and FeNO levels, suggesting that this in 2025 latest-available antibody with the broadest label was also used for patients who did not qualify for other biologics because they did not exhibit markedly increased type 2 inflammatory markers. In addition, these patients also had the lowest average GINA control status and ACT score, highlighting a discordance between clinical disease severity and type 2 biomarkers in a subpopulation of patients with severe asthma. 

Furthermore, we observed that after switching the initial biologic therapy to a different one, improvements in treatment response were achieved in all analyzed switch constellations, reflecting less exacerbations, less oral cortisone use, and better symptom control. In a relevant proportion of patients, remission was achieved after a switch, which is the ultimate goal of modern, targeted asthma therapy. On the one hand, these results reflect the increasing therapeutical options in asthma biologics, with improving fit of antibodies to the respective phenotype. On the other hand, we deduce that when response to the initial antibody is insufficient, switching to a different one is a rational next step with a good chance of resulting in better disease control and even remission. 

As the GAN registry has been ongoing since 2011, our data provides an important overview of the history of biologic prescription in severe asthma in a country where all currently available biologics in this indication are covered by the statutory health insurance if EMA licensing criteria are fulfilled. Thus, the choice of initial biologic in the registry possibly better reflects phenotypic and medical considerations than in countries where not all biologics have been available or reimbursement is limited to even more severe disease characteristics than the licensing criteria. For example, data from the UK registry from 2022 and 2023 show that > 99% of patients received an anti-IL5/anti-IL5R or anti-IgE drug as initial therapy, whereas < 1% had an anti-IL4R therapy, possibly due to regulation by insurance coverage [18, 19]. 

Other real-world studies have investigated the effects of switching biologic therapy in severe asthma, comparing their effectiveness for disease control and found benefits of switching in case the of insufficient outcome. However, these studies usually focused on one specific switch constellation, e.g., from omalizumab to mepolizumab [20, 21, 22, 23, 24, 25], from omalizumab or mepolizumab to benralizumab [26, 27, 28, 29, 30, 31], or from either of the previous ones to dupilumab [32, 33, 34, 35]. Besides, the most recently developed anti-TSLP antibody tezepelumab was not considered in these studies [36, 37, 38]. Thus, our data demonstrates the complexity of real-world switching practice in a whole registry and regarding every possible switch constellation. 

Our study has some limitations. First, it relies on observational data from a registry that requires active inclusion by the treating physician and consent of the patient. Thus, even if the registry aims to include patients with severe asthma in Germany without any restriction, it is subject to possible selection bias. Second, follow-up data was not available for all switch constellations but was generally limited to the more frequently observed ones and relatively small numbers for individual switch constellations. Of note, switches occurred mostly from an older biologic to a newer one, and rarely vice versa, likely reflecting the gain of experience in selecting the right biologic for the right patient. In addition, no data for follow-up after switch to anti-TSLP was available due to the recent availability of this drug. Therefore, our results can only reflect a contemporary picture of the practice and outcomes after switch of asthma biologics. Importantly, our analysis should not be mistaken as a comparison between different drugs, as only those patients with an insufficient outcome were switched to a different biologic, whereas the majority responded to the initial therapy [10] and did not need to switch. Furthermore, our study assumes that switching generally occurred due to insufficient treatment response to the first biologic; yet, adverse reactions might also be a reason for switching, but this data was not available for our analysis. 

However, our real-world study of the large GAN severe asthma cohort adds important data by analyzing the patterns and effects of switch configurations to a different biologic treatment in severe asthma accounting for all six currently available antibodies. Its results encourage the concept of adjustment of therapy to find the best fitted medication to the patient phenotype and thus reach an optimal treatment response, ideally remission. 

We conclude that phenotyping of patients with severe asthma is an important factor determining the initial choice of biologic therapy in real-world practice. Choice and switch of biologic have evolved over time influenced by availability of different drugs, further improving outcomes by adjustment to the best fitted therapy. 

## Acknowledgment 

We would like to thank all GAN centers and investigators for their collaboration and the patients for their participation. 

All GAN centers are listed here: https://germanasthmanet.de/centers/.

## Authors’ contributions 

AL and KM conceived the study, prepared figures and wrote the manuscript 

AH analyzed the data including statistical analysis and prepared figures. 

All authors (RB, CM, JB RE, EH, MI, MJ, FK, OS, CS, DS, HS, CT, SK) provided patient data from their severe asthma centers, critically revised and approved the manuscript. 

## Funding 

This study was supported by the German Asthma Net (GAN). GAN is financially supported by the AstraZeneca, Chiesi, GSK, and Sanofi; these sponsors did not have any role in the design of the study, data collection, data analysis, or the writing of the manuscript. 

## Conflict of interest 

AL: received speaker and/or advisory fees from AstraZeneca and Sanofi. RB: reports grants to Mainz University and personal fees from Boehringer Ingelheim, GSK, Novartis, and Roche as well as personal fees from AstraZeneca, Celltrion, Chiesi, Cipla, Sanofi, and Teva, all outside the submitted work. CM: received speaker and/or advisory fees from AstraZeneca, and Sanofi. JB: personal fees for consultancy and lectures from AbbVie, AstraZeneca, Biogen, Boehringer-Ingelheim, Bristol Myers Squibb, Ferrer, Gossamer Bio, Johnson & Johnson, Novartis, Pliant, Pulmovant, Sanofi-Genzyme, and United Therapeutics, outside the submitted work. RE: no conflicts of interest. EH: is a member of advisory boards for ALK, Sanofi, AImmune, AstraZeneca, and GSK. He consulted or received speaker’s fees from ALK, AImmune, AstraZeneca, Bencard, Boehringer, GSK, HAL, Milupa, Novartis, Sanofi, and Stallergenes. AH: no conflicts of interest. MI: reports lectures fees from AstraZeneca, Bayer, Berlin-Chemie, Boehringer Ingelheim, Chiesi, CSL-Behring, GSK, Menarini, MSD, Novartis, Roche, Sanofi, and Thermofischer and advisory board fees from Alk-Pharma, AstraZeneca, Berlin-Chemie, Boehringer Ingelheim, Chiesi, CSL-Behring, GSK, Novartis, and Sanofi, all outside the submitted work. MJ: no conflicts of interest. FK: no conflicts of interest. OS: received speaker and advisory fees from AstraZeneca, Chiesi, and Sanofi. CS: reports consultancy fees from AstraZeneca; and payment or honoraria for lectures, presentations, manuscript writing, or educational events from AstraZeneca, Novartis, and Sanofi. DS: received speaker and advisory fees from AstraZeneca, GSK, and Sanofi. HS: received speaker and/or advisory fees from AstraZeneca, Chiesi, GSK, and Sanofi. CT: no conflicts of interest. SK: reports speaker fees from AstraZeneca, GSK, Novartis, Sanofi, all outside the submitted work. KM received speaker and/ or advisory fees from AstraZeneca, Chiesi, GSK, Novartis, Sanofi. 

**Table 1.** Patient characteristics at baseline visit (V0) by first initiated biologic therapy for severe asthma in the GAN registry 2011 – 2024.

**Baseline characteristics**		**Anti-IgE (n = 634)**	**Anti-IL5R (n = 693)**	**Anti-IL5 (n = 670)**	**Anti-IL4R (n = 417)**	**Anti-TSLP (n = 235)**	p-value*
Sex	N	623	693	664	417	235	
	Female	363 (58.3%)	359 (51.8%)	371 (55.9%)	219 (52.5%)	120 (51.1%)	0.0965
Age at inclusion (years)	N	632	692	668	417	234	
	Mean (SD)	49.8 (14.5)	58.3 (12.8)	57.9 (13.3)	54.5 (13.1)	57.7 (13.6)	**< 0.0001**
BMI (kg/m^2^)	N	633	687	665	415	232	
	Mean (SD)	27.4 (5.8)	27.8 (6.1)	27.1 (5.7)	27.9 (5.9)	28.1 (6.0)	0.0528
Age at asthma diagnosis (years)	N	424	499	470	288	130	
	Mean (SD)	26.5 (18.8)	41.0 (19.0)	41.2 (17.5)	37.3 (19.0)	34.1 (19.4)	**< 0.0001**
Smoking habits	N	634	691	669	417	235	
	Non-smoker	347 (54.7%)	361 (52.2%)	350 (52.3%)	218 (52.3%)	126 (53.6%)	0.5661
	Smoker	30 (4.7%)	19 (2.7%)	23 (3.4%)	15 (3.6%)	11 (4.7%)	
	Former smoker	257 (40.5%)	311 (45.0%)	296 (44.2%)	184 (44.1%)	98 (41.7%)	
Asthma phenotype	N	607	647	627	378	213	
	Predominantly allergic	461 (75.9%)	185 (28.6%)	157 (25.0%)	139 (36.8%)	77 (36.2%)	< 0.0001
	Non-allergic	28 (4.6%)	275 (42.5%)	304 (48.5%)	108 (28.6%)	54 (25.4%)	
	Mixed form	118 (19.4%)	187 (28.9%)	166 (26.5%)	131 (34.7%)	82 (38.5%)	
Any allergic comorbidities†	N	626	353	444	388	217	
	Existent	498 (79.6%)	172 (48.7%)	229 (51.6%)	237 (61.1%)	119 (54.8%)	**< 0.0001**
Chronic sinusitis	N	634	693	669	415	235	
	Existent	251 (39.6%)	338 (48.8%)	360 (53.8%)	208 (50.1%)	60 (25.5%)	**< 0.0001**
Nasal polyps	N	383	693	570	415	235	
	Existent	98 (25.6%) N = 383	267 (38.5%)	231 (40.5%) N = 570	185 (44.6%)	45 (19.1%)	**< 0.0001**
Exacerbations in the last 12 months	N	610	686	657	416	234	
	None	143 (23.4%)	157 (22.9%)	179 (27.2%)	87 (20.9%)	38 (16.2%)	**0.0001**
	Unknown	34 (5.6%)	44 (6.4%)	43 (6.5%)	34 (8.2%)	31 (13.2%)	
	1x/year	122 (20.0%)	89 (13.0%)	96 (14.6%)	58 (13.9%)	32 (13.7%)	
	> 1x/year, but < 1x/month	266 (43.6%)	327 (47.7%)	276 (42.0%)	194 (46.6%)	111 (47.4%)	
	≥ 1x/month	45 (7.4%)	69 (10.1%)	63 (9.6%)	43 (10.3%)	22 (9.4%)	
GINA control status	N	634	693	669	417	235	
	Controlled	134 (21.1%)	116 (16.7%)	152 (22.7%)	85 (20.4%)	16 (6.8%)	**< 0.0001**
	Partly controlled	172 (27.1%)	119 (17.2%)	153 (22.9%)	59 (14.1%)	53 (22.6%)	
	Uncontrolled	328 (51.7%)	458 (66.1%)	364 (54.4%)	273 (65.5%)	166 (70.6%)	
ACT (points)	N	565	626	597	378	216	
	Median (IQR)	16.0 (11 – 20)	14 (10 – 19)	16 (12 – 22)	14 (11 – 20)	14.0 (9 – 17)	**< 0.0001**
FEV_1_ [L]	N	600	660	644	406	223	
	Mean (SD)	2.23 (0.89)	2.01 (0.80)	2.05 (0.86)	2.15 (0.81)	2.04 (0.85)	**< 0.0001**
FEV_1_ [%]	N	594	659	636	406	217	
	Mean (SD)	69.83 (22.14)	66.45 (21.82)	69.17 (22.29)	67.90 (21.54)	64.56 (20.82)	**0.0037**
FeNO [ppb]	N	307	394	395	298	149	
	Median (IQR)	26.0 (14.0 – 48.0)	39.5 (23.0 – 69.0)	38.0 (23.0 – 65.0)	35.0 (17.0 – 68.0)	24.0 (13.0 – 50.0)	**< 0.0001**
Blood eosinophil count [/µL]	N	313	408	433	259	134	
	Median (IQR)	205.4 (104.5 – 361.2)	432.7 (173.3 – 793.4)	214.2 (61.3 – 560.0)	300.0 (144.2 – 540.5)	159.0 (80.0 – 320.0)	**< 0.0001**
Total IgE [IU/mL]	N	231	190	185	81	113	
	Median	419 (176; 759)	136.5 (57; 360)	152 (54; 354)	144 (34; 829)	128 (48; 301)	**< 0.0001**
Maintenance OCS	N	634	693	670	417	235	
	Yes	182 (28.7%)	234 (33.8%)	237 (35.4%)	109 (26.1%)	56 (23.8%)	**0.0004**

*p-value: difference between all groups. For comparing categorical values χ^2^-test was used, for comparing continuous values Kruskal-Wallis test was used; ^†^allergic comorbidities: allergic rhinitis/rhinoconjunctivitis, food allergy, atopic dermatitis, other allergic comorbidities. Statistically significant results with p < 0.05 were highlighted in bold. ACT = asthma control test; anti-IgE = anti-immunoglobulin E; anti-IL5 = anti-interleukin-5; anti-IL5R = anti-interleukin-5 receptor; anti-IL4R = anti-interleukin-4 alpha receptor; anti-TSLP = anti-thymic stromal lymphopoietin; FeNO = fractional exhaled nitric oxide (expressed in parts per billion, ppb); FEV_1_ = forced expiratory volume in 1 second; GAN = German Asthma Net; IQR = interquartile range; OCS = oral corticosteroids; SD = standard deviation.

**Table 2.** Switch constellations between first and second initiated biologic therapy for severe asthma in the GAN registry 2011 – 2024.

	**Initial biologic**					
**Second biologic**	**anti-IgE (n = 634)**	**anti-IL5R (n = 693)**	**anti-IL5 (n = 670)**	**anti-IL4R (n = 417)**	**anti-TSLP (n = 235)**	**Total**
No switch documented	540 (85.2%)	588 (84.5%)	533 (79.6%)	379 (90.9%)	231 (98.3%)	2,271 (85.7%
Anti-IL5R	15 (2.4%)	–	64 (9.6%)	14 (3.4%)	–	93 (3.5%)
Anti-IL5	28 (4.4%)	18 (2.6%)	–	3 (0.7%)	–	49 (1.8%)
Anti-IL4R	28 (4.4%)	59 (8.5%)	53 (7.9%)	–	3 (1.3%)	143 (5.4%)
Anti-IgE	–	2 (0.3%)	4 (0.6%)	3 (0.7%)	1 (0.4%)	10 (0.4%)
Anti-TSLP	23 (3.6%)	26 (3.8%)	16 (2.4%)	18 (4.3%)	–	83 (3.1%)
Total who switched	94 (14.8%)	105 (15.2%)	137 (20.4%)	38 (9.1%)	4 (1.7%)	378 (14.3%)
Duration of initial biologic before switch (months) (median, IQR)	19.87 (11.90 – 46.36)	22.36 (12.16 – 33.54)	24.23 (12.15 – 42.44)	14.43 (7.84 – 32.36)	10.90 (8.59 – 12.51)	

GAN = German Asthma Net; anti-IgE = anti-immunoglobulin E; anti-IL5 = anti-interleukin-5; anti-IL5R = anti-interleukin-5 receptor; anti-IL4R = anti-interleukin-4 alpha receptor; anti-TSLP = anti-thymic stromal lymphopoietin; IQR = interquartile range.

**Table 3.** Biologic asthma response score (BARS) after switching to a different biologic therapy in severe asthma in the GAN registry 2011 – 2024.

**Item**		**Anti-IgE – anti-IL5**	**Anti-IgE – anti-IL4R**	**Anti-IL5 – anti-IL5R**	**Anti-IL5 – anti-IL4R**	**Anti-IL5R – anti-IL4R**
BARS I (mean)	N	8	17	42	24	34
	Mean (SD)	1.11 (0.66)	1.07 (0.61)	1.05 (0.76)	1.13 (0.79)	1.16 (0.69)
	Median	1.17	1.00	1.00	1.00	1.33
	IQR	1.00 – 1.33	0.50 – 1.50	0.50 – 1.83	0.50 – 2.00	0.67 – 1.67
	Missing values	2	7	14	7	13
BARS I (grouped)	N	8	17	42	24	34
	No response	1 (16.7%)	1 (10.0%)	6 (21.4%)	3 (17.6%)	4 (19.0%)
	Intermediate response	4 (66.7%)	5 (50.0%)	10 (35.7%)	6 (35.3%)	7 (33.3%)
	Good response	1 (16.7%)	4 (40.0%)	12 (42.9%)	8 (47.1%)	10 (47.6%)
	Missing values	2	7	14	7	13
BARS II (mean)	N	8	17	42	24	34
	Mean (SD)	0.87 (0.54)	1.02 (0.49)	0.89 (0.69)	0.94 (0.80)	1.02 (0.60)
	Median	1.00	1.00	0.88	1.00	1.25
	IQR	0.75 – 1.25	0.67 – 1.33	0.00 – 1.67	0.00 – 1.67	0.50 – 1.50
	Missing values	3	8	20	13	19
BARS II (grouped)	N	8	17	42	24	34
	No response	1 (20.0%)	2 (22.2%)	6 (27.3%)	4 (36.4%)	3 (20.0%)
	Intermediate response	4 (80.0%)	5 (55.6%)	10 (45.5%)	3 (27.3%)	8 (53.3%)
	Good response	–	2 (22.2%)	6 (27.3%)	4 (36.4%)	4 (26.7%)
	Missing values	3	8	20	13	19

BARS I = mean score taking into account exacerbations, oral corticosteroid therapy, and ACT score; BARS II = mean score taking into account exacerbations, oral corticosteroid therapy, ACT score, and FEV_1_. ACT = asthma control test; anti-IgE = anti-immunoglobulin E; anti-IL5 = anti-interleukin-5; anti-IL5R = anti-interleukin-5 receptor; anti-IL4R = anti-interleukin-4 alpha receptor; anti-TSLP = anti-thymic stromal lymphopoietin; BARS = Biologic Asthma Response Score [10]; GAN = German Asthma Net; SD = standard deviation; IQR = interquartile range.

**Table 4.** Remission after switching to a different biologic therapy in the GAN registry 2011 – 2024.

**Item**		**Anti-IgE – anti-IL5**	**Anti-IgE – anti-IL4R**	**Anti-IL5 – anti-IL5R**	**Anti-IL5 – anti-IL4R**	**Anti-IL5R – anti-IL4R**
Exacerbation last 12 months at post visit	N	8	17	42	24	34
	None	6 (75.0%)	12 (70.6%)	28 (66.7%)	15 (62.5%)	22 (64.7%)
	At least 1	2 (25.0%)	5 (29.4%)	14 (33.3%)	9 (37.5%)	12 (35.3%)
Maintenance OCS at post visit	N	8	17	42	24	34
	No	3 (37.5%)	16 (94.1%)	29 (69.0%)	22 (91.7%)	28 (82.4%)
	Yes	5 (62.5%)	1 (5.9%)	13 (31.0%)	2 (8.3%)	6 (17.6%)
ACT points at post visit	N	8	17	42	24	34
	< 20 ACT points	2 (25.0%)	9 (52.9%)	17 (40.5%)	10 (41.7%)	17 (50.0%)
	≥ 20 ACT points	6 (75.0%)	8 (47.1%)	25 (59.5%)	14 (58.3%)	17 (50.0%)
Difference to baseline in FEV_1_ [L]	N	8	17	42	24	34
	< 0.1 L	5 (62.5%)	8 (47.1%)	23 (62.2%)	13 (59.1%)	19 (63.3%)
	≥ 0.1 L	3 (37.5%)	9 (52.9%)	14 (37.8%)	9 (40.9%)	11 (36.7%)
	Missing values	–	–	5	2	4
Remission I	N	8	17	42	24	34
	No remission	6 (75.0%)	12 (70.6%)	26 (61.9%)	13 (54.2%)	23 (67.6%)
	Remission	2 (25.0%)	5 (29.4%)	16 (38.1%)	11 (45.8%)	11 (32.4%)
Remission II	N	8	17	42	24	34
	No remission	7 (87.5%)	16 (94.1%)	33 (89.2%)	17 (77.3%)	28 (93.3%)
	Remission	1 (12.5%)	1 (5.9%)	4 (10.8%)	5 (22.7%)	2 (6.7%)
	Missing values	–	–	5	2	4

Remission I: No exacerbations in the previous 12 months, no OCS use; and ACT ≥ 20 points. Remission II: No exacerbations in the previous 12 months, no OCS use, ACT ≥ 20 points; and FEV_1_ improvement of ≥ 100 mL. OCS = oral corticosteroids; ACT = asthma control test; FEV_1_ = forced expiratory volume in 1 second; L = liters; GAN = German Asthma Net.

## Supplemental material

Supplemental material Supplemental Tables
