# Supplementary material for: Choosing and switching biologics for patients with severe asthma: Real-world data from the German Asthma Net (GAN)
Source: Allergol Select. 2026 Jul 20;10:158–69. doi: 10.5414/ALX02636E (PMC13409342; doi:10.5414/ALX02636E)
Supplement: Supplemental material [file allergologieselect-10-158-S01.pdf]

Supplementary table E1: Antibody switch constellations available for follow-up analysis in the GAN registry 2011-2024.

Initial AB	Switch AB	Number of cases	% of total†	Cases with FU visits
anti-IL5R	anti-IL5	18	4.8	
	anti-IL4	59	15.6	43
	anti-IgE	2	0.5	
	anti-TSLP	26	6.9	
anti-IL5	anti-IL5/R	64	16.9	54
	anti-IL4	53	14.0	35
	anti-IgE	4	1.1	
	anti-TSLP	16	4.2	
anti-IL4R	anti-IL5/R	14	3.7	
	anti-IL5	3	0.8	
	anti-IgE	3	0.8	
	anti-TSLP	18	4.8	
anti-IgE	anti-IL5/R	15	4.0	
	anti-IL5	28	7.4	15
	anti-IL4	28	7.4	23
	anti-TSLP	23	6.1	
anti-TSLP	anti-IL4	3	0.8	
	anti-IgE	1	0.3	
Total		378		170

FU: follow-up available 1 year, 2 years or 4 months after switch of biologic therapy.

†percentage of total number of switches (N=378)

Anti-IgE: anti-immunglobulin E, Anti-IL5: anti-interleukin-5, Anti-IL5R: anti-interleukin-5 receptor, Anti-IL4R: anti-interleukin-4 receptor, Anti-TSLP: anti-thymic stromal lymphopoietin. GAN: German Asthma Net.

Supplementary table E2: Number of exacerbations in the last 12 months before and after switch of biologic therapy in the GAN registry 2011-2024.

		Initial AB		Switch AB						
Switch		First visit	Last visit	First visit	After 4 months	After 1 year	After 2 years	Post	Diff.: Post-First	Signed Rang Test
anti-IL5 - anti-IL5R (n=54)	N	37	32	40	5	37	25	40	40	
	Mean (SD)	2.35 (2.31)	1.66 (1.60)	0.95 (1.22)	1.80 (2.68)	0.38 (0.79)	0.60 (1.15)	0.43 (0.87)	-0.53 (1.22)	
	Median	2.00	1.50	0.50	0.00	0.00	0.00	0.00	0.00	**p=0.0124**
	IQR	1.00 - 3.00	0.00 - 2.50	0.00 - 2.00	0.00 - 3.00	0.00 - 0.00	0.00 - 1.00	0.00 - 0.50	-1.00 - 0.00	
anti-IL5 - anti-IL4R (n=35)	N	22	22	26	4	25	11	26	26	
	Mean (SD)	4.18 (8.30)	1.45 (2.04)	1.19 (2.40)	0.75 (1.50)	0.60 (1.29)	0.91 (1.45)	0.58 (1.27)	-0.62 (2.65)	
	Median	2.50	0.50	0.50	0.00	0.00	0.00	0.00	0.00	p=0.2852
	IQR	0.00 - 4.00	0.00 - 2.00	0.00 - 1.00	0.00 - 1.50	0.00 - 1.00	0.00 - 2.00	0.00 - 1.00	-1.00 - 0.00	
anti-IL5R - anti-IL4R (n=43)	N	30	27	34	5	23	18	34	34	
	Mean (SD)	3.70 (3.60)	2.63 (3.44)	1.76 (2.77)	0.80 (1.30)	0.30 (0.93)	0.50 (1.04)	0.44 (1.05)	-1.32 (2.85)	
	Median	3.00	1.00	0.50	0.00	0.00	0.00	0.00	0.00	**p=0.0062**
	IQR	1.00 - 6.00	0.00 - 5.00	0.00 - 3.00	0.00 - 1.00	0.00 - 0.00	0.00 - 1.00	0.00 - 0.00	-3.00 - 0.00	
anti-IgE - anti-IL5 (n=15)	N	11	12	12	.	12	9	12	12	
	Mean (SD)	2.82 (3.34)	2.50 (3.29)	0.92 (0.90)		0.83 (1.27)	1.00 (1.66)	0.83 (1.27)	-0.08 (1.31)	
	Median	2.00	2.00	1.00	.	0.00	0.00	0.00	0.00	p=1.0000
	IQR	1.00 - 4.00	0.50 - 3.00	0.00 - 2.00		0.00 - 1.50	0.00 - 1.00	0.00 - 1.50	-1.00 - 0.50	
anti-IgE - anti-IL4R (n=23)	N	13	12	16	2	14	10	16	16	
	Mean (SD)	3.00 (3.16)	2.58 (4.25)	0.88 (1.31)	0.00 (0.00)	0.07 (0.27)	0.20 (0.42)	0.06 (0.25)	-0.81 (1.38)	
	Median	3.00	1.00	0.00	0.00	0.00	0.00	0.00	0.00	p=0.0625
	IQR	1.00 - 4.00	0.00 - 3.50	0.00 - 2.00	0.00 - 0.00	0.00 - 0.00	0.00 - 0.00	0.00 - 0.00	-2.00 - 0.00	

AB: antibody therapy. Anti-IgE: anti-immunglobulin E, Anti-IL5: anti-interleukin-5, Anti-IL5R: anti-interleukin-5 receptor, Anti-IL4R: anti-interleukin-4 receptor, Anti-TSLP: anti-thymic stromal lymphopoietin. GAN: German Asthma Net. IQR: interquartile range. SD: standard deviation.

Supplementary table E3: Change in GINA control status after switch of biologic therapy in the GAN registry 2011-2024.

		Switch group				
	**Changes to first visit**	**anti-IL5 - anti-IL5R**	**anti-IL5 - anti-IL4R**	**anti-IL5R - anti-IL4R**	**anti-IgE - anti-IL5**	**anti-IgE - anti-IL4R**
GINA control status	N	54	35	43	15	23
	2 levels improvement	4 (7.4%)	3 (8.6%)	5 (11.6%)	-	3 (13.0%)
	1 level improvement	18 (33.3%)	7 (20.0%)	12 (27.9%)	5 (33.3%)	2 (8.7%)
	no change	22 (40.7%)	23 (65.7%)	22 (51.2%)	9 (60.0%)	17 (73.9%)
	1 level deterioration	9 (16.7%)	2 (5.7%)	4 (9.3%)	1 (6.7%)	1 (4.3%)
	2 levels deterioration	1 (1.9%)	-	-	-	-
p-value (signed rank test)		**0.0266**	**0.0225**	**0.0010**	0.2188	0.1250

Anti-IgE: anti-immunglobulin E, Anti-IL5: anti-interleukin-5, Anti-IL5R: anti-interleukin-5 receptor, Anti-IL4R: anti-interleukin-4 receptor, Anti-TSLP: anti-thymic stromal lymphopoietin. GAN: German Asthma Net.

Supplementary table E4: Change in ACT score after switch of biologic therapy in the GAN registry 2011-2024.

		Initial AB		Switch AB						
**Switch**		**First visit**	**Last visit**	**First visit**	**After 4 months**	**After 1 year**	**After 2 years**	**Post**	**Diff.: Post-First**	**Signed Rang Test**
anti-IL5 - anti-IL5R (n=54)	N	46	47	49	12	43	25	49	49	
	Mean (SD)	15.63 (4.91)	16.53 (4.96)	18.49 (4.71)	19.08 (6.20)	19.02 (5.41)	18.28 (4.55)	18.73 (5.57)	0.24 (4.23)	
	Median	15.00	16.00	19.00	21.50	20.00	19.00	20.00	0.00	p=0.6100
	IQR	11.00 - 20.00	12.00 - 21.00	15.00 - 22.00	13.00 - 24.00	18.00 - 23.00	15.00 - 22.00	16.00 - 23.00	-2.00 - 3.00	
anti-IL5 - anti-IL4R (n=35)	N	25	28	28	10	24	12	28	28	
	Mean (SD)	14.28 (6.02)	16.25 (5.39)	17.25 (5.71)	18.70 (5.72)	17.96 (5.92)	17.42 (5.92)	17.89 (5.88)	0.64 (4.58)	
	Median	14.00	16.50	18.00	22.00	19.50	19.00	19.50	0.00	p=0.5330
	IQR	9.00 - 18.00	11.00 - 20.00	13.00 - 22.00	14.00 - 22.00	13.00 - 23.00	11.00 - 22.00	13.00 - 22.50	-1.00 - 2.50	
anti-IL5R - anti-IL4R (n=43)	N	33	35	36	14	27	17	36	36	
	Mean (SD)	13.36 (5.59)	14.66 (6.25)	16.47 (6.48)	16.43 (5.56)	19.41 (5.29)	20.18 (4.23)	18.72 (5.44)	2.25 (4.90)	
	Median	13.00	14.00	17.00	15.00	21.00	21.00	19.00	1.00	**p=0.0181**
	IQR	9.00 - 17.00	10.00 - 20.00	10.50 - 22.50	14.00 - 21.00	16.00 - 24.00	16.00 - 24.00	15.00 - 24.00	-1.00 - 4.50	
anti-IgE - anti-IL5 (n=15)	N	9	9	10	.	9	6	10	10	
	Mean (SD)	18.00 (5.17)	18.78 (6.00)	21.60 (3.66)		21.67 (4.12)	22.50 (3.15)	21.40 (3.98)	-0.20 (1.40)	
	Median	20.00	20.00	23.00	.	23.00	24.00	22.50	0.00	p=0.8281
	IQR	16.00 - 21.00	16.00 - 23.00	21.00 - 24.00		21.00 - 24.00	19.00 - 25.00	19.00 - 24.00	-1.00 - 1.00	
anti-IgE - anti-IL4R (n=23)	N	17	19	20	6	18	11	20	20	
	Mean (SD)	13.82 (5.63)	14.79 (4.35)	15.70 (4.99)	15.17 (7.39)	18.44 (6.09)	17.00 (5.66)	18.05 (5.91)	2.35 (5.43)	
	Median	13.00	16.00	16.00	13.50	21.00	17.00	20.00	1.00	**p=0.0369**
	IQR	10.00 - 18.00	12.00 - 17.00	12.50 - 20.00	10.00 - 24.00	15.00 - 23.00	13.00 - 24.00	14.00 - 23.00	-0.50 - 4.00	

AB: antibody therapy. Anti-IgE: anti-immunglobulin E, Anti-IL5: anti-interleukin-5, Anti-IL5R: anti-interleukin-5 receptor, Anti-IL4R: anti-interleukin-4 receptor, Anti-TSLP: anti-thymic stromal lymphopoietin. GAN: German Asthma Net. IQR: interquartile range. SD: standard deviation.

Supplementary table E5: Change in FEV_1 (litres) after switch of biologic therapy in the GAN registry 2011-2024.

		Initial AB		Switch AB						
Switch		First visit	Last visit	First visit	After 4 months	After 1 year	After 2 years	Post	Diff.: Post-First	Signed Rang Test
anti-IL5 - anti-IL5R (n=54)	N	45	45	46	12	43	27	46	46	
	Mean (SD)	2.02 (0.88)	2.00 (0.85)	2.01 (0.93)	2.04 (0.94)	2.09 (0.93)	2.25 (1.05)	2.08 (0.91)	0.08 (0.42)	
	Median	1.76	1.70	1.77	1.75	1.94	1.99	1.88	0.00	p=0.4497
	IQR	1.36 - 2.65	1.36 - 2.65	1.34 - 2.52	1.38 - 2.69	1.46 - 2.53	1.36 - 2.84	1.46 - 2.53	-0.24 - 0.33	
anti-IL5 - anti-IL4R (n=35)	N	33	34	34	16	28	16	34	34	
	Mean (SD)	1.97 (0.80)	2.01 (0.79)	2.05 (0.84)	2.20 (0.89)	2.22 (0.90)	2.18 (0.76)	2.21 (0.91)	0.16 (0.51)	
	Median	1.89	2.10	1.83	2.02	2.09	2.05	2.09	0.05	p=0.1854
	IQR	1.30 - 2.44	1.35 - 2.39	1.36 - 2.47	1.54 - 2.80	1.66 - 2.76	1.64 - 2.43	1.62 - 2.80	-0.12 - 0.33	
anti-IL5R - anti-IL4R (n=43)	N	34	35	37	17	26	16	37	37	
	Mean (SD)	1.87 (0.67)	1.97 (0.80)	2.10 (0.89)	2.35 (0.97)	2.23 (0.86)	2.25 (0.91)	2.25 (0.80)	0.15 (0.40)	
	Median	1.76	1.82	1.97	2.30	2.33	2.20	2.34	0.04	p=0.1626
	IQR	1.35 - 2.25	1.37 - 2.47	1.51 - 2.69	1.57 - 2.76	1.41 - 2.65	1.43 - 2.87	1.57 - 2.65	-0.14 - 0.38	
anti-IgE - anti-IL5 (n=15)	N	12	12	13	1	13	9	13	13	
	Mean (SD)	2.21 (1.12)	2.02 (0.93)	2.33 (1.01)	2.98 (.)	2.39 (0.86)	2.33 (0.82)	2.39 (0.86)	0.06 (0.36)	
	Median	1.98	1.84	2.67	2.98	2.58	2.61	2.58	0.04	p=0.6709
	IQR	1.10 - 3.26	1.03 - 2.90	1.32 - 3.06	2.98 - 2.98	2.13 - 3.10	1.53 - 3.05	2.13 - 3.10	-0.20 - 0.35	
anti-IgE - anti-IL4R (n=23)	N	20	21	21	6	19	12	21	21	
	Mean (SD)	2.28 (0.95)	2.08 (0.90)	2.26 (0.90)	1.97 (0.71)	2.34 (0.85)	2.26 (1.08)	2.39 (0.85)	0.13 (0.40)	
	Median	2.10	1.90	2.05	2.07	2.23	1.80	2.23	0.02	p=0.2123
	IQR	1.64 - 2.97	1.48 - 2.38	1.65 - 3.07	1.43 - 2.17	1.78 - 2.92	1.52 - 3.43	1.88 - 2.92	-0.07 - 0.23	

AB: antibody therapy. Anti-IgE: anti-immunglobulin E, Anti-IL5: anti-interleukin-5, Anti-IL5R: anti-interleukin-5 receptor, Anti-IL4R: anti-interleukin-4 receptor, Anti-TSLP: anti-thymic stromal lymphopoietin. GAN: German Asthma Net. IQR: interquartile range. SD: standard deviation.

Supplementary table E6: Change in FEV_1 (%predicted) after switch of biologic therapy in the GAN registry 2011-2024.

		Initial AB		Switch AB						
Switch		First visit	Last visit	First visit	After 4 months	After 1 year	After 2 years	Post	Diff.: Post-First	Signed Rang Test
anti-IL5 - anti-IL5R (n=54)	N	44	44	46	12	43	27	46	46	
	Mean (SD)	65.1 (21.5)	65.5 (21.8)	67.2 (26.3)	67.8 (23.2)	71.1 (24.5)	76.5 (23.4)	70.8 (24.3)	3.6 (13.9)	
	Median	67.0	65.0	66.8	69.0	72.0	77.0	71.5	2.0	p=0.1246
	IQR	49.5 - 83.5	49.0 - 83.5	43.0 - 90.4	50.5 - 88.5	55.0 - 90.0	58.0 - 97.0	55.0 - 90.0	-4.0 - 12.0	
anti-IL5 - anti-IL4R (n=35)	N	32	33	33	16	27	15	33	33	
	Mean (SD)	65.6 (21.4)	66.1 (18.3)	68.6 (21.4)	72.8 (18.6)	75.3 (19.2)	71.0 (16.6)	74.5 (18.7)	5.9 (13.5)	
	Median	67.7	61.7	72.0	73.5	78.0	74.0	73.0	2.0	**p=0.0254**
	IQR	47.5 - 79.5	57.0 - 79.0	50.0 - 87.0	62.0 - 89.5	64.0 - 85.0	57.0 - 79.0	64.0 - 85.0	-2.0 - 14.0	
anti-IL5R - anti-IL4R (n=43)	N	34	35	37	17	26	16	37	37	
	Mean (SD)	63.9 (19.7)	67.2 (23.6)	71.8 (23.5)	80.4 (21.2)	75.8 (23.9)	74.3 (22.9)	77.6 (21.5)	5.9 (14.0)	
	Median	63.0	64.0	75.0	85.0	80.0	77.9	83.0	2.0	p=0.0603
	IQR	51.0 - 76.0	50.0 - 83.0	56.0 - 95.0	71.0 - 90.0	57.0 - 95.0	54.5 - 94.0	67.0 - 93.0	-3.0 - 15.0	
anti-IgE - anti-IL5 (n=15)	N	12	12	13	1	13	9	13	13	
	Mean (SD)	66.1 (26.8)	61.5 (20.6)	69.2 (24.9)	91.0 (.)	74.9 (24.6)	73.1 (22.4)	74.9 (24.6)	5.7 (16.4)	
	Median	58.5	59.7	73.0	91.0	75.1	74.0	75.1	2.0	p=0.4126
	IQR	49.0 - 94.0	48.0 - 79.2	49.4 - 89.0	91.0 - 91.0	67.0 - 93.6	68.0 - 82.0	67.0 - 93.6	-4.8 - 9.1	
anti-IgE - anti-IL4R (n=23)	N	18	21	21	6	19	12	21	21	
	Mean (SD)	67.4 (21.2)	62.0 (18.1)	69.0 (20.0)	62.2 (10.9)	72.7 (18.0)	68.1 (18.8)	72.5 (17.5)	3.5 (10.5)	
	Median	67.5	58.0	69.0	60.0	73.0	63.0	73.0	2.0	p=0.1988
	IQR	49.0 - 83.0	52.0 - 73.0	55.0 - 86.0	57.0 - 64.0	64.0 - 92.0	52.5 - 84.5	64.0 - 83.0	-4.0 - 7.0	

AB: antibody therapy. Anti-IgE: anti-immunglobulin E, Anti-IL5: anti-interleukin-5, Anti-IL5R: anti-interleukin-5 receptor, Anti-IL4R: anti-interleukin-4 receptor, Anti-TSLP: anti-thymic stromal lymphopoietin. GAN: German Asthma Net. IQR: interquartile range. SD: standard deviation.

Supplementary table E7: Change in FeNO (ppb) after switch of biologic therapy in the GAN registry 2011-2024.

		Initial AB		Switch AB						
Switch		First visit	Last visit	First visit	After 4 months	After 1 year	After 2 years	Post	Diff.: Post-First	Signed Rang Test
anti-IL5 - anti-IL5R (n=54)	N	15	18	25	4	21	13	25	25	
	Mean (SD)	58.0 (59.4)	46.2 (50.2)	58.9 (70.0)	19.5 (10.1)	66.5 (54.1)	34.4 (28.1)	59.5 (52.2)	0.6 (43.9)	
	Median	46.0	42.0	32.0	20.5	48.0	26.0	40.0	2.0	p=0.5915
	IQR	28.0 - 53.0	14.0 - 54.0	22.0 - 58.0	11.5 - 27.5	23.0 - 84.0	15.0 - 45.0	23.0 - 81.0	-8.0 - 18.0	
anti-IL5 - anti-IL4R (n=35)	N	14	18	19	9	15	5	19	19	
	Mean (SD)	70.4 (70.5)	61.2 (37.3)	64.2 (47.6)	32.2 (20.7)	26.5 (13.7)	43.0 (32.0)	29.6 (18.1)	-34.6 (45.4)	
	Median	53.0	54.5	44.0	25.0	24.0	27.0	24.0	-20.0	**p=0.0021**
	IQR	31.0 - 73.0	35.0 - 78.0	26.0 - 96.0	18.0 - 38.0	18.0 - 31.0	26.0 - 68.0	19.0 - 31.0	-65.0 - -2.0	
anti-IL5R - anti-IL4R (n=43)	N	17	19	21	8	13	5	21	21	
	Mean (SD)	59.8 (54.2)	68.6 (56.2)	60.5 (45.6)	30.9 (11.7)	34.7 (15.4)	28.2 (11.6)	31.9 (13.9)	-28.7 (44.9)	
	Median	46.0	60.0	66.0	26.5	32.0	29.0	27.0	-16.0	**p=0.0080**
	IQR	27.0 - 66.0	26.0 - 80.0	26.0 - 79.0	22.0 - 41.5	25.0 - 48.0	20.0 - 30.0	20.0 - 39.0	-55.0 - 2.0	
anti-IgE - anti-IL5 (n=15)	N	5	4	5	.	5	3	5	5	
	Mean (SD)	74.8 (58.5)	83.8 (58.8)	61.2 (64.6)		60.2 (57.4)	61.3 (44.2)	60.2 (57.4)	-1.0 (27.4)	
	Median	49.0	74.0	24.0	.	20.0	41.0	20.0	-1.0	p=1.0000
	IQR	28.0 - 115.0	36.5 - 131.0	18.0 - 94.0		18.0 - 118.0	31.0 - 112.0	18.0 - 118.0	-4.0 - 8.0	
anti-IgE - anti-IL4R (n=23)	N	5	11	13	5	9	5	13	13	
	Mean (SD)	51.8 (16.6)	45.5 (29.1)	41.7 (32.0)	21.4 (6.1)	45.0 (36.1)	15.4 (8.7)	35.4 (33.4)	-6.3 (13.8)	
	Median	48.0	36.0	31.0	18.0	35.0	17.0	23.0	-5.0	p=0.1118
	IQR	47.0 - 48.0	30.0 - 65.0	28.0 - 57.0	17.0 - 28.0	19.0 - 59.0	7.0 - 21.0	16.0 - 44.0	-16.0 - 0.0	

FeNO: fractional exhaled nitric oxide, expressed in parts per billion (ppb). AB: antibody therapy. Anti-IgE: anti-immunglobulin E, Anti-IL5: anti-interleukin-5, Anti-IL5R: anti-interleukin-5 receptor, Anti-IL4R: anti-interleukin-4 receptor, Anti-TSLP: anti-thymic stromal lymphopoietin. GAN: German Asthma Net. IQR: interquartile range. SD: standard deviation.

Supplementary table E8: Change in absolute blood eosinophil count (/µl) after switch of biologic therapy in the GAN registry 2011-2024.

		Initial AB		Switch AB						
**Switch**		**First visit**	**Last visit**	**First visit**	**After 4 months**	**After 1 year**	**After 2 years**	**Post**	**Diff.: Post-First**	**Signed Rang Test**
anti-IL5 - anti-IL5R (n=54)	N	9	7	12	5	11	1	12	12	
	Mean (SD)	517.5 (505.9)	192.8 (342.0)	89.5 (104.4)	23.3 (33.3)	30.0 (43.8)	15.6 (.)	34.4 (44.4)	-55.2 (104.8)	
	Median	413.1	59.0	60.7	10.0	11.8	15.6	11.9	-15.0	p=0.0674
	IQR	67.0 - 967.2	50.0 - 106.0	7.7 - 133.5	10.0 - 14.4	10.0 - 28.1	15.6 - 15.6	10.0 - 43.8	-50.7 - 2.4	
anti-IL5 - anti-IL4R (n=35)	N	15	14	19	10	15	8	19	19	
	Mean (SD)	347.9 (451.9)	99.2 (41.9)	332.0 (434.7)	555.2 (337.6)	436.3 (329.1)	655.6 (652.0)	449.0 (308.2)	116.9 (474.9)	
	Median	275.0	104.0	166.4	470.5	351.4	290.8	351.4	85.7	p=0.2101
	IQR	61.3 - 416.1	61.3 - 124.8	70.0 - 341.0	350.0 - 808.4	165.0 - 545.6	244.0 -1113.3	175.0 - 573.3	-79.4 - 317.0	
anti-IL5R - anti-IL4R (n=43)	N	13	13	17	7	11	4	17	17	
	Mean (SD)	354.2 (283.4)	130.9 (152.6)	326.1 (408.4)	718.6 (483.2)	620.3 (423.1)	344.5 (224.1)	609.8 (399.5)	283.7 (576.3)	
	Median	376.7	75.2	104.0	829.4	550.0	332.5	482.2	232.1	**p=0.0395**
	IQR	84.3 - 575.0	33.9 - 127.2	58.1 - 588.0	359.3 -1249.0	286.0 - 941.6	162.7 - 526.3	288.8 - 869.0	72.6 - 473.9	
anti-IgE - anti-IL5 (n=15)	N	5	5	5	.	5	4	5	5	
	Mean (SD)	600.9 (411.0)	579.9 (470.0)	103.1 (72.0)		112.1 (61.3)	80.0 (60.4)	112.1 (61.3)	9.0 (23.6)	
	Median	550.0	550.0	79.5	.	87.4	52.9	87.4	7.9	p=0.6250
	IQR	418.1 - 702.2	155.1 - 852.5	57.2 - 120.0		83.7 - 100.0	45.1 - 115.0	83.7 - 100.0	0.0 - 12.2	
anti-IgE - anti-IL4R (n=23)	N	7	4	10	4	7	6	10	10	
	Mean (SD)	234.8 (162.8)	378.2 (177.5)	264.3 (169.4)	199.8 (77.8)	450.4 (428.1)	291.1 (357.7)	382.8 (368.7)	118.5 (264.3)	
	Median	214.0	437.1	203.3	188.5	330.4	154.1	276.6	49.7	p=0.3223
	IQR	127.4 - 378.2	252.8 - 503.7	150.0 - 438.0	140.2 - 259.4	110.0 -1020.3	96.0 - 253.2	122.0 - 349.8	-28.0 - 157.4	

AB: antibody therapy. Anti-IgE: anti-immunglobulin E, Anti-IL5: anti-interleukin-5, Anti-IL5R: anti-interleukin-5 receptor, Anti-IL4R: anti-interleukin-4 receptor, Anti-TSLP: anti-thymic stromal lymphopoietin. GAN: German Asthma Net. IQR: interquartile range. SD: standard deviation.
